# WSES guidelines for emergency repair of complicated abdominal wall hernias

**DOI:** 10.1186/1749-7922-8-50

**Published:** 2013-12-01

**Authors:** Massimo Sartelli, Federico Coccolini, Gabrielle H van Ramshorst, Giampiero Campanelli, Vincenzo Mandalà, Luca Ansaloni, Ernest E Moore, Andrew Peitzman, George Velmahos, Fredrick Alan Moore, Ari Leppaniemi, Clay Cothren Burlew, Walter Biffl, Kaoru Koike, Yoram Kluger, Gustavo P Fraga, Carlos A Ordonez, Salomone Di Saverio, Ferdinando Agresta, Boris Sakakushev, Igor Gerych, Imtiaz Wani, Michael D Kelly, Carlos Augusto Gomes, Mario Paulo Faro, Korhan Taviloglu, Zaza Demetrashvili, Jae Gil Lee, Nereo Vettoretto, Gianluca Guercioni, Cristian Tranà, Yunfeng Cui, Kenneth YY Kok, Wagih M Ghnnam, Ashraf El-Sayed Abbas, Norio Sato, Sanjay Marwah, Muthukumaran Rangarajan, Offir Ben-Ishay, Abdul Rashid K Adesunkanmi, Helmut Alfredo Segovia Lohse, Jakub Kenig, Stefano Mandalà, Andrea Patrizi, Rodolfo Scibé, Fausto Catena

**Affiliations:** 1 Department of Surgery, Macerata Hospital, Macerata, Italy; 2 General Surgery Department, Papa Giovanni XXIII hospital, Bergamo, Italy; 3 Department of Surgery, Red Cross Hospital Beverwijk, Erasmus University Medical Center, Rotterdam, Netherlands; 4 Department of Surgical Science, Istituto clinic Sant’Ambrogio, Milan, Italy; 5 Department of Surgery, Buccheri La Ferla Hospital, Palermo, Italy; 6 Department of Surgery, Denver Health Medical Center, Denver, CO, USA; 7 Department of Surgery, University of Pittsburgh School of Medicine, Pittsburgh, USA; 8 Harvard Medical School, Division of Trauma, Emergency Surgery and Surgical Critical Care Massachusetts General Hospital, Boston, MA, USA; 9 Department of Surgery, University of Florida, Gainesville, Florida, USA; 10 Department of Abdominal Surgery, University Hospital Meilahti, Helsinki, Finland; 11 Department of Primary Care & Emergency Medicine, Kyoto University Graduate School of Medicine, Kyoto, Japan; 12 Department of General Surgery, Rambam Health Care Campus, Haifa, Israel; 13 Division of Trauma Surgery, Hospital de Clinicas -, School of Medical Sciences, University of Campinas, Campinas, Brazil; 14 Department of Surgery, Fundacion Valle del Lili, Universidad del Valle, Cali, Colombia; 15 Department of Surgery, Maggiore Hospital, Bologna, Italy; 16 Department of Surgery, Adria Civil Hospital, Adria, RO, Italy; 17 First Clinic of General Surgery, University Hospital /UMBAL/ St George Plovdiv, Plovdiv, Bulgaria; 18 Department of Surgery 1, Lviv Regional Hospital, Danylo Halytsky Lviv National Medical University, Lviv, Ukraine; 19 Department of Surgery, Sheri-Kashmir Institute of Medical Sciences, Srinagar, India; 20 Griffith Base Hospital, Griffith, NSW, Australia; 21 Faculdade de Ciências Médicas e da Saúde de Juiz de Fora (SUPREMA), Federal University of Juiz de Fora (UFJF), Juiz de Fora, MG, Brazil; 22 Department of General Surgery, Trauma and Emergency Surgery Division, ABC Medical School, Santo André, SP, Brazil; 23 Department of General Surgery, Istanbul Doctor’s Center, Istanbul, Turkey; 24 Department of Surgery, Tbilisi State Medical University, Tbilisi, Georgia; 25 Department of Surgery, Yonsei University College of Medicine, Seoul, Korea; 26 Laparoscopic Surgical Unit, M. Mellini Hospital, Chiari, BS, Italy; 27 Department of Surgery, Mazzoni Hospital, Ascoli Piceno, Italy; 28 Department of Surgery, Tianjin Nankai Hospital, Nankai Clinical School of Medicine, Tianjin Medical University, Tianjin, China; 29 Department of Surgery, Ripas Hospital, Bandar Seri Begawan, Brunei; 30 Department of Surgery Mansoura, Faculty of Medicine, Mansoura University, Mansoura, Egypt; 31 Department of Surgery, Pt BDS Post-graduate Institute of Medical Sciences, Rohtak, India; 32 Department of Laparoscopic Surgery, GEM Hospital & Research Center, Coimbatore, India; 33 Department of Surgery, College of Health Sciences, Obafemi Awolowo University Hospital, Ile-Ife, Nigeria; 34 II Cátedra de Clínica Quirúrgica, Hospital de Clínicas, Facultad de Ciencias Médicas, Universidad Nacional de Asuncion, San Lorenzo, Paraguay; 35 3rd Department of General Surgery, Jagiellonian University Collegium Medium, Krakow, Poland; 36 Department of Surgery, G. Giglio Hospital Cefalù, Palermo, Italy; 37 Emergency Surgery, Maggiore Parma Hospital, Parma, Italy

## Abstract

Emergency repair of complicated abdominal hernias is associated with poor prognosis and a high rate of post-operative complications.

A World Society of Emergency Surgery (WSES) Consensus Conference was held in Bergamo in July 2013, during the 2^nd^ Congress of the World Society of Emergency Surgery with the goal of defining recommendations for emergency repair of abdominal wall hernias in adults. This document represents the executive summary of the consensus conference approved by a WSES expert panel.

## Introduction

A large number of abdominal hernias require emergency surgery. However, these procedures are associated with poor prognoses and a higher rate of post-operative complications [1].

A World Society of Emergency Surgery (WSES) Consensus Conference was held in Bergamo on July 2013, during the 2^nd^ Congress of the World Society of Emergency Surgery with the goal of defining recommendations for emergency repair of abdominal wall hernias in adults. This document represents the executive summary of the consensus conference approved by a WSES expert panel.

Abdominal hernias may be classified as groin hernias (femoral and inguinal) and ventral hernias (umbilical, epigastric, spigelian and incisional).

An incarcerated hernia may be defined as a hernia in which the contents have become irreducible due to a narrow opening in the abdominal wall or adhesions within the cavity. Intestinal obstruction can complicate an incarcerated hernia. In contrast, a strangulated hernia is one in which the blood supply to the contents of the hernia (eg omentum, bowel) s becomes compromised [2].

Strangulated hernias remain a significant challenge, as they are sometimes difficult to diagnose purely by physical examination yet require urgent surgical intervention. Early surgical intervention of a strangulated hernia with obstruction is crucial as delayed diagnosis can lead to bowel resection with longer recovery and its attendant complications. Strangulated hernias can have serious deleterious effects such as, bowel obstruction, bacterial translocation, and intestinal wall necrosis (potentially resulting in bowel perforation). It poses a significant risk to emergency hernia repair, as there is an increased incidence of surgical field contamination, leading to high rates of post-operative infection and probably recurrence.

Bacteria inherently colonize all surgical wounds, but only a fraction of these contaminates ultimately lead to infection. In most patients infection does not occur because innate host defences are able to eliminate microbes at the surgical site. However, there is some evidence that the implantation of foreign materials, such as prosthetic mesh, may lead to a decreased threshold for infection [3].

While many factors can influence surgical wound healing and post-operative infection, bacterial burden is the most significant risk factor. Wounds are classified according to the likelihood and degree of wound contamination at the time of operation. Classifications include: clean wounds, clean-contaminated wounds, contaminated wounds, and dirty or infected wounds [4].

The pathogens involved in an infection depend on the type of surgery. In an aseptic surgical procedure, *Staphylococcus aureus* is a common source of infection, either from the patient’s own skin flora or surrounding environment. Surgeons can minimize the risk of infection and associated complications by routinely employing site-specific spectrum antibiotic prophylaxis.

In clean-contaminated, contaminated, and dirty surgical procedures, the polymicrobial aerobic and anaerobic flora closely resemble the normal endogenous microflora of the gastrointestinal (GI) tract and are the most frequently observed pathogens. The contaminating pathogens in GI surgery include gram-negative bacilli (e.g., *Escherichia coli*) and gram-positive microbes, such as enterococci and anaerobic organisms. A classification scheme has been demonstrated in multiple studies to predict the relative probability that a given wound will become infected [5,6].

Several studies show clear advantages of mesh use in elective cases, where infection should be uncommon. Mesh significantly reduces the rate of hernia recurrence yet is easy to use and has low complication rates. On the other hand, few studies have investigated the outcome of mesh use in an emergency setting, where there is often surgical field contamination due to bowel involvement [7,8].

The use of biological mesh has many advantages, including a decreased immune response mounted against the foreign body, as well as decreased incidence of fistulae formation, fibrosis, and erosions.

There is, however, a paucity of high quality evidence on the superiority of biological mesh and there remains a significant price premium with their use [9].

Recommendation guidelines are evaluated according to the Grading of Recommendations Assessment, Development, and Evaluation (GRADE), a hierarchical, evidence-based rubric [10,11] summarized in Table 1, which is a guideline used to assess the strength of recommendations.

**Table 1:** **Grading of Recommendations Assessment, Development, and Evaluation (GRADE) from Guyatt and colleagues**[10,11]

**Grade of recommendation**	**Clarity of risk/benefit**	**Quality of supporting evidence**	**Implications**
1A			
Strong recommendation, high-quality evidence	Benefits clearly outweigh risk and burdens, or vice versa	RCTs without important limitations or overwhelming evidence from observational studies	Strong recommendation, applies to most patients in most circumstances without reservation
1B			
Strong recommendation, moderate-quality evidence	Benefits clearly outweigh risk and burdens, or vice versa	RCTs with important limitations (inconsistent results, methodological flaws, indirect analyses or imprecise conclusions) or exceptionally strong evidence from observational studies	Strong recommendation, applies to most patients in most circumstances without reservation
1C			
Strong recommendation, low-quality or very low-quality evidence	Benefits clearly outweigh risk and burdens, or vice versa	Observational studies or case series	Strong recommendation but subject to change when higher quality evidence becomes available
2A			
Weak recommendation, high-quality evidence	Benefits closely balanced with risks and burden	RCTs without important limitations or overwhelming evidence from observational studies	Weak recommendation, best action may differ depending on the patient, treatment circumstances, or social values
2B			
Weak recommendation, moderate-quality evidence	Benefits closely balanced with risks and burden	RCTs with important limitations (inconsistent results, methodological flaws, indirect or imprecise) or exceptionally strong evidence from observational studies	Weak recommendation, best action may differ depending on the patient, treatment circumstances, or social values
2C			
Weak recommendation, Low-quality or very low-quality evidence	Uncertainty in the estimates of benefits, risks, and burden; benefits, risk, and burden may be closely balanced	Observational studies or case series	Very weak recommendation; alternative treatments may be equally reasonable and merit consideration

## Recommendations

### Timing of intervention

Patients should undergo emergency hernia repair immediately when intestinal strangulation is suspected (grade 1C recommendation).

Systemic inflammatory response syndrome (SIRS) signs, contrast-enhanced CT findings as well as lactate, CPK and D-dimer levels are predictive of bowel strangulation (grade 1C recommendation).

Unfortunately, morbidity and mortality rates remain high for patients who undergo emergency repair of abdominal hernias. Early diagnosis of strangulated obstruction maybe difficult, and delayed diagnosis can lead to septic complications. However, in the case of suspected bowel strangulation the benefits outweigh the risks of surgery and patients should undergo immediate surgical intervention.

A recent study performed by Martínez-Serrano et al. prospectively analyzed morbidity and mortality rates following emergency hernia repair [12]. The study population included 244 patients with complicated abdominal wall hernias requiring surgical repair. In this study, the patients were treated according to standardized protocols with detailed actions to be taken during the pre-, intra-, and post-operative periods. Clinical outcomes were compared retroactively to that of 402 patients who had undergone similar procedures before the development and implementation of the protocols outlined in the study. Results showed higher rates of mortality in patients with acute complication as their first hernia-related symptom and whose treatment was delayed for more than 24 hours. Thus, the authors concluded that early detection of complicated abdominal hernias may be the best means of reducing the rate of mortality [12].

In 2007, Derici et al. published a retrospective study using univariate and multivariate analysis to investigate factors affecting morbidity and mortality rates in cases of incarcerated abdominal wall hernias [13]. Using univariate analysis, results showed that symptomatic periods lasting longer than 8 hours, the presence of comorbid disease, high American Society of Anesthesiology (ASA) scores, the use of general anesthesia, the presence of strangulation, and the presence of necrosis significantly affect morbidity rates. In contrast, advanced age, the presence of comorbid diseases, high ASA scores, the presence of strangulation, the presence of necrosis, and hernia repair with graft were found to significantly affect mortality rates by univariate analysis; the presence of necrosis, however, was the only factor that appeared to significantly affect mortality rates based on multivariate analysis [10].

A retrospective study was recently published evaluating the risk factors associated with bowel resection and treatment outcome in patients with incarcerated groin hernias [14].

The study analyzed 182 adult patients with incarcerated groin hernias who underwent emergency hernia repair in the 10-year period from January 1999 to June 2009. Of these patients, bowel resection was required in 15.4% of cases (28/182). A logistic regression model identified three independent risk factors for bowel resection: lack of health insurance (odds ratio [OR], 5, P = 0.005), obvious peritonitis (OR, 11.52, P = 0.019), and femoral hernia (OR, 8.31, P < 0.001) [14].

Many authors reported that early detection of progression from an incarcerated hernia to a strangulated hernia is difficult to achieve by either clinical or laboratory means, which presents a large challenge in early diagnosis [15-17]. Signs of SIRS including fever, tachycardia, and leukocytosis, as well as abdominal wall rigidity, are considered common indicators of strangulated obstruction. However, an investigation by Sarr et al. demonstrated that the combination of four classic signs of strangulation – continuous abdominal pain, fever, tachycardia, and leukocytosis – could not distinguish strangulated from simple obstructions [16]. Furthermore, Shatilla et al. reported a low incidence of these classical findings and stated that their presence indicated an advanced stage of strangulation, which would be of limited value for early diagnosis [16]. In 2006, Tsumura et al. published a retrospective study investigating SIRS as a predictor of strangulated small bowel obstruction. Multivariate analysis revealed that the presence of SIRS alongside abdominal muscle guarding was independently predictive of strangulated small bowel obstruction [18].

Among possible diagnostic tests, serum creatinine phosphokinase (CPK) appears to be a relatively reliable indicator of early intestinal strangulation [19,20]. Icoz et al. published a prospective study investigating the relevance of serum D-dimer measurement as a potential diagnostic indicator of strangulated intestinal hernia. The authors concluded that D-dimer assays should be performed on patients presenting with intestinal emergencies to better evaluate and predict ischemic events. Despite having low specificity, elevated D-dimer levels measured upon admission were found to correlate strongly with intestinal ischemia [21].

In 2012 an interesting retrospective study examining whether various laboratory parameters could predict viability of strangulation in patients with bowel obstruction was published. Forty patients diagnosed with bowel strangulation operated within 72 hours of the start of symptoms were included in the study. Lactate level was the only laboratory parameter significantly associated with viability (P < 0.01, Mann-Whitney test). Other laboratory data did not show statistically significant associations. The Authors concluded that arterial blood lactate level (2.0 mmol/L or greater) was a useful predictor of nonviable bowel strangulation [22].

Early diagnostic methods to detect bowel strangulation have advanced substantially following the development and refinement of radiological techniques, such as Computed Tomography (CT) scanning [23]. Jancelewicz et al. recently published a retrospective analysis demonstrating that CT findings of reduced wall enhancement were the most significant independent predictor of bowel strangulation, with 56% sensitivity and 94% specificity. By contrast, elevated white blood cell (WBC) count and guarding on physical examination were only moderately predictive. It should be noted, however, that an elevated WBC was the only variable found to be independently predictive of bowel strangulation in patients with small bowel obstruction [24].

### Laparoscopic approach

Repair of incarcerated hernias – both ventral and groin – may be performed with a laparoscopic approach (grade 1C recommendation).

Recent prospective studies and recent guidelines [25-31] have focused on the laparoscopic approach to hernia repair in an elective setting.

By contrast, few studies have focused on the laparoscopic approach to hernia repair in an emergency setting. In 2004, Landau et al. published a retrospective study investigating the use of laparoscopy in the repair of incarcerated incisional and ventral hernias. The authors argued that laparoscopic repair was feasible and could be safely used to treat patients presenting with incarcerated incisional and ventral hernias [32].

Another retrospective study published in 2008 investigated the role of laparoscopy in the management of incarcerated (non-reducible) ventral hernias. The authors concluded that laparoscopic repair of ventral abdominal wall hernias could be safely performed with low subsequent complication rates, even in the event of an incarcerated hernia. Careful bowel reduction with adhesiolysis and mesh repair in an uncontaminated abdomen (without inadvertent enterotomy) using a 5-cm mesh overlap was an important factor predictive of successful clinical outcome [33].

In 2009, another retrospective study was published investigating laparoscopic techniques used to treat incisional hernias in an emergency setting. The results of this series also demonstrated the feasibility of laparoscopic surgery to treat incarcerated incisional hernias in an emergency setting [34].

Additionally, a systematic literature review performed in 2009 identified articles reporting on laparoscopic treatment, reduction, and repair of incarcerated or strangulated inguinal hernias from 1989 to 2008. It included seven articles on this topic, reporting on 328 cases treated with total extraperitoneal (TEP) or transabdominal preperitoneal (TAPP) repair. Laparoscopy can also be used to resect bowel, if necessary, or to repair an occult contralateral hernia, present in 11.2–50% of cases. The Authors concluded that the laparoscopic repair is a feasible procedure with acceptable results; however, its efficacy needs to be studied further, ideally with larger, multicenter randomized controlled trials [35]

In 2007 a series of patients with large irreducible groin hernias (omentoceles), treated by laparoscopy without conversions, was published. The Authors described a technique to facilitate complete removal of the the hernia contents. A laparoscopic transperitoneal repair for large irreducible scrotal hernias removing as much omentum as possible was performed. Then a small groin incision was made to excise the adherent omentum from the distal sac [36].

Hernioscopy is a mixed laparoscopic–open surgical technique for incarcerated inguinal hernias. Specifically, it is effective in evaluating the viability of the herniated loop, thus avoiding unnecessary laparotomy [37]. A prospective randomized study in 2009 aimed to evaluate the impact of hernia sac laparoscopy on the morbidity and mortality of cases with a spontaneous reduction of the strangulated hernia content before the assessment of its viability. Ninety-five patients were randomly assigned to 2 groups: group A (21 patients managed using hernia sac laparoscopy) and group B (20 patients managed without laparoscopy). The median hospital stay was 28 hours for group A and 34 hours for group B. Four patients of group B had major complications, whereas there was none observed in the group A. Two unnecessary laparotomies and 2 deaths occurred in group B. The authors concluded that hernia sac laparoscopy seems to be an accurate and safe method of preventing unnecessary laparotomy and in high-risk patients it contributes to decreased morbidity [38].

### Emergency hernia repair in “clean surgical field”

The choice of technique repair is based on the contamination of the surgical field, the size of the hernia and the experience of the surgeon.

Prosthetic repair with synthetic mesh is recommended for patients with intestinal incarceration and no signs of intestinal strangulation or concurrent bowel resection (clean surgical field) (grade 1A recommendation).

The increased likelihood of surgical site infection may suggest additive risk for permanent synthetic mesh repair (grade 1C recommendation).

Primary suture repair as an elective hernia-related procedure can increase the risk of recurrence, thereby leading to subsequent follow-up surgery. This is the case in both ventral and inguinal abdominal wall hernias. Numerous studies have demonstrated the advantages of mesh use in clean, sterile cases; such advantages include ease of placement, low long-term complication rates, and reduction of recurrence for incisional hernias [39-42]. For patients with intestinal incarceration and no signs of intestinal strangulation or concurrent bowel resection, the surgical field is presumed clean and the infectious risk for synthetic mesh is low. The absence of intestinal wall ischemia renders patients less predisposed to bacterial translocation, and there is a low risk of need for concurrent bowel resection, which leads to contamination of the surgical field. However, this has not been proven for cases of acute irreducible hernias.

Researchers have published a variety of small-scale studies comparing mesh use to suture repair in the treatment of acute irreducible hernias [43-46]. In 2011, Nieuwenhuizen et al. published a retrospective study investigating the use of mesh in acute hernia-related procedures. A total of 203 patients were identified for the study: 76 inguinal, 52 umbilical, 39 incisional, 14 epigastric, 14 femoral, 5 trocar, and 3 spigelian hernias. For purposes of statistical analysis, epigastric, femoral, trocar, and spigelian hernia patients were pooled together due to their small individual group sizes. One patient was excluded from the analysis because the hernia was not ultimately corrected during surgery. In all, 99 hernias were repaired using mesh compared to 103 primary suture repairs. Additionally, univariate analysis demonstrated that female patients (*P* = 0.007), overweight patients (*P* = 0.016), patients with an umbilical hernia (*P* = 0.01), and patients who had undergone bowel resection (*P* = 0.015) featured significantly higher rates of wound infection. By contrast, the type of repair (i.e. primary suture vs. mesh), the use of antibiotic prophylaxis, ASA class, and patient age did not appear to share any statistically significant relationships with post-operative rates of surgical site infection. Based on logistic regression analysis, only bowel resection (*P* = 0.020) appeared to correlate significantly with post-operative surgical site infection [47].

An increased likelihood for surgical site infection may suggest additive risk for permanent synthetic mesh repair [48-50]. In a recent multicenter cohort study, patients who underwent incisional hernia repair during other concomitant intra-abdominal procedures experienced greater than 6-fold increases in the risk of subsequent mesh removal. Of the 1,071 mesh repairs retrospectively analyzed during the 4-year period from 1998 to 2002, 5.1% (55/1,071) underwent mesh removal at a median time of 7.3 months (interquartile range 1.4-22.2) following incisional hernia repair with permanent mesh prosthesis. Infection was the most common reason for mesh removal, accounting for 69% of cases. No statistically significant differences were observed based on the method of surgical repair. After adjusting for covariates, both same-site concomitant surgery (hazard ratio [HR] = 6.3) and post-operative surgical site infection (HR = 6.5) were associated with mesh removal [51].

### Emergency hernia repair in “potentially contaminated surgical field”

For patients with intestinal strangulation and/or concurrent bowel resection (potentially contaminated surgical field), direct suture is recommended when the hernia defect in question is small. Synthetic mesh repair may be performed, but with caution. Biological meshes may be a valid option but merit detailed cost-benefit analysis (grade 2C recommendation).

Many studies discuss and advocate the use of prosthetic mesh in clean surgical fields. However, the use of prosthetic grafts in potentially-contaminated and contaminated settings is seldom described. Despite discrepancies in data and conflicting reports, prosthetic materials are not generally recommended for abdominal hernia repair in contaminated settings. Most studies on the subject do not focus on emergency repair, and as such, their results are of limited value. According to many researchers, the use of mesh is strongly discouraged in potentially contaminated surgical fields.

One study analyzed and compared post-operative outcome following ventral hernia repair using prosthetic mesh in clean-contaminated and contaminated wounds [52]. All patients of U.S. hospitals participating in the National Surgical Quality Improvement Program (NSQIP) who were admitted for mesh-mediated ventral hernia repair in the 5-year period from January 1, 2005, to April 4, 2010, were included in the study. Compared to clean cases, clean-contaminated cases featured a significantly greater likelihood of wound disruption, pneumonia, and sepsis as well as superficial, deep, and ventral surgical site infections (SSIs). Both clean-contaminated and contaminated mesh-mediated cases featured an increased risk of septic shock (5.82% and 26.74%, respectively) and ventilator use lasting longer than 48 hours (5.59% and 26.76%, respectively). Clean-contaminated cases of mesh-mediated ventral hernia repair also featured a significantly increased odds ratio for complications (2.52) [52].

In a recent study, Xourafas et al. examined the impact of mesh use on ventral hernia repairs with simultaneous bowel resections attributable to either cancer or bowel occlusion. Researchers found a significantly higher incidence of post-operative infection in patients with prosthetic mesh compared to those without mesh. According to multivariate regression analysis, prosthetic mesh use was the only significant risk factor irrespective of other variables such as drain use, defect size, or type of bowel resection [53]. By contrast, other researchers have asserted that prosthetic repair of abdominal hernias can be safely performed alongside simultaneous colonic operations. Such joint procedures, they argue, exhibit acceptable rates of infectious complications and recurrence, and consequently, they maintain that there is insufficient evidence to advocate the avoidance of prosthetic mesh in potentially contaminated fields, assuming that the appropriate technique is used [54,55].

In 2000 Mandalà et al. published a series of patients with incisional hernias treated with nonabsorbable prostheses and associated visceral surgery. The low incidence of suppurative complications, with neither removal of the patch nor recurrences in the short term, showed that nonabsorbable mesh repair in potentially contaminated fields was safe [56].

Studies by Vix et al., Birolini et al., and Geisler et al. report wound-related morbidity rates of 10.6%, 20%, and 7%, respectively, following mesh use in both clean-contaminated and contaminated procedures [57-59]. A different study by Campanelli et al. analyzed ten prosthetic hernia repairs in potentially contaminated fields and reported no major or minor complications after a 21-month follow-up period [60].

Recently a study by Carbonell et al. [61] investigated Open ventral hernia repairs performed with polypropylene mesh in the retro-rectus position in clean-contaminated and contaminated fields. The 30-day surgical site infection rate was 7.1% for clean-contaminated cases; for contaminated cases the 30-day surgical site infection rate was 19.0%.

It should be noted, however, that most of these studies did not focus on emergency repair of incarcerated hernias.

A study by Kelly et al. reported a 21% infection rate in a series of emergency and elective incisional hernia repairs [62]. A study by Davies et al. focused exclusively on a subset of hernia cases in which patients presented with an obstructed bowel and required emergency surgery. This study found high rates of infection in patients requiring emergency repair for all types of abdominal hernias [63]. A retrospective multivariate analysis by Nieuwenhuizen et al. revealed bowel resection to be a major factor associated with wound infection, but that other clinical ramifications of the procedure were relatively rare [47]. A recently published retrospective analysis of emergency repair of incarcerated incisional hernias with simultaneous bowel obstruction in potentially contaminated fields demonstrated that the use of permanent prosthetic mesh in these surgeries was associated with high rates of wound infection. No infections occurred in patients whose surgical wounds were left open to granulate [64].

In 2013 a prospective study to present a 7-year experience with the use of prosthetic mesh repair in the management of the acutely incarcerated and/or strangulated ventral hernias was published. The hernia was para-umbilical in 71 patients (89%), epigastric in 6 patients (8%) and incisional in 3 patients (4%). Eighteen patients (23%) had recurrent hernias. Resection-anastomosis of non-viable small intestine was performed in 18 patients (23%) and was not regarded as a contraindication for prosthetic repair [65].

Biological mesh prosthetics are most commonly used in infected fields involving large, complex abdominal wall hernia repairs. The use of biological mesh, which becomes vascularized and remodelled into autologous tissue after implantation, may offer a low-morbidity alternative to prosthetic mesh products in these complex settings, with good results also in immunocompromised patients [66]. The use of biological materials in clinical practice has led to innovative methods of treating abdominal wall defects in contaminated surgical fields.

Many retrospective studies have explored the promising role of biological mesh in contaminated fields, but most of these investigations did not focus on emergency repair of incarcerated hernias [67-87].

Although biologic mesh in these situations is safe, long-term durability has still not been demonstrated [88]. A study by Catena et al. published in 2007 focused on complicated incisional hernia repair using mesh prosthetics made of porcine dermal collagen (PDC). Incisional hernioplasty using PDC grafts was found to be a safe and efficient approach to difficult cases complicated by potential contamination [82].

A recent literature review by Coccolini et al. covered the use of biological meshes for abdominal reconstruction in emergency and elective setting in transplanted patients, and reported a complication rate of 9.4% [85].

By incorporating biological mesh, surgeons hope to provide a collagen-based extracellular matrix scaffold by which host fibroblasts can induce angiogenesis and deposit new collagen. The non-synthetic material of biological mesh makes it less susceptible to infection, and several biological grafts are available in the current market. Their classification is based on the species of origin (allogenic or xenogenic), the type of collagen matrix utilized (dermis, pericardium, or intestinal submucosa), the decellularization process, the presence or absence of cross-linkage, temperature-related storage requirements, and the use of rehydration [86].

On the basis of either the presence or not of the cross-linking, biological prosthesis are divided into two subgroups: the partially remodeling (cross-linked) and the completely remodeling ones (not cross-linked). Thanks to the presence of additional linkages the partially remodeling ones resist better and for a longer period to mechanical stress [66].

Coccolini et al. recently published the results of the first 193 patients of the Italian Register of Biological Prosthesis (IRBP) [87]. This prospective multi-centre study, suggests the usefulness, versatility and ease of using biological prosthesis in many different situations, including clean or contaminated surgical fields. Despite the lack of a cohesive body of evidence, published studies on biological mesh suggest that cross-linked mesh prosthetics have the lowest failure rate in potentially contaminated and outright infected fields. This trend should be investigated further by means of large, prospective, randomized studies [89].

Recently a critical review of biologic mesh use in ventral hernia repairs under contaminated field was published. All literature reviews found in medline database supported biologic mesh use, especially in the setting of contaminated fields, but the primary literature included in these reviews consisted entirely of case series and case reports with low levels of evidence [90]. To better guide surgeons, prospective, randomized trials should be undertaken to evaluate the short- and long-term outcomes associated with biological meshes under the various surgical wound classifications [91].

### Emergency hernia repair in “contaminated-dirty surgical field”

For stable patients with strangulated obstruction and peritonitis by bowel perforation (contaminated-dirty surgical field) direct tissue suture is recommended when the hernia defect is small; in the events that direct tissue suture is not possible, biological mesh repair may be suggested (grade 2C recommendation). The choice between a cross-linked or a non cross-linked biological mesh should be evaluated depending on the defect size and degree of contamination (grade 2C recommendation).

If biological mesh is not available, both polyglactin mesh repair and open management with delayed repair may be a viable alternative (grade 2C recommendation).

For unstable patients (those experiencing severe sepsis or septic shock), open management is recommended to prevent abdominal compartment syndrome; intra-abdominal pressure may be measured intra-operatively (grade 2C recommendation).

Following stabilization of the patient, surgeons should attempt early, definitive closure of the abdomen. Primary fascial closure may be possible when there is minimal risk of excessive tension or recurrence of intra-abdominal hypertension (IAH) (grade 2C recommendation).

In the event that early, definitive fascial closure is not possible, surgeons must resort to progressive closure performed incrementally each time the patient returns for a subsequent procedure. Cross-linked biological meshes may be considered an option in abdominal wall reconstruction (grade 2C recommendation).

In cases of bacterial peritonitis, patients must undergo contaminated surgical intervention, which means that the surgical field is infected and the risk of surgical site infection is very high. As mentioned earlier, the use of biological materials in clinical practice has led to innovative methods of treating abdominal wall defects in contaminated surgical fields, although there is still insufficient level of high-quality evidence on their value, and there is still a very huge price difference between the synthetic and biological meshes (9).

Some authors investigated the use of absorbable prosthetic materials [86]. However, the use of absorbable prosthesis exposes the patient to an inevitable hernia recurrence. These meshes, once implanted, initiate an inflammatory reaction that, through a hydrolytic reaction, removes and digests the implanted prosthetic material completely. In this case, the high risk of hernia recurrence is explained by the complete dissolution of the prosthetic support [92].

Patients with strangulated obstruction and peritonitis caused by bowel perforation are often considered critically ill due to septic complications; further, they may experience high intra-operative intra-abdominal pressure, which can lead to abdominal compartment syndrome. Although intra-abdominal hypertension has been known to cause physiological perturbation since the early 19th century, its clinical implications have only recently been recognized in patients sustaining intra-abdominal trauma. Such hypertension may be the underlying cause of increased pulmonary pressures, reduced cardiac output, splanchnic hypoperfusion, and oliguria. In summary, this clinical condition is known as abdominal compartment syndrome. Abdominal compartment syndrome results from shock and resuscitation yielding ischemic reperfusion-related injury. Cellular damage results from ischemia, subsequent cellular membrane dysfunction, and intra- and extra-cellular edema. This capillary leak results in massive edema of local tissues, most notably those of the intestines. Prophylactic treatment to avoid abdominal compartment syndrome involves refraining from abdominal closure when fascial approximation becomes problematic due to excessive tension [93].

Intestinal strangulation can lead to increased intra-abdominal pressure, and ultimately, to abdominal compartment syndrome. A study published by Beltran et al. examined 81 consecutive unselected patients presenting with complicated hernias and intestinal obstruction. The researchers measured intra-abdominal pressure using the intra-vesicular pressure method, and these serial measurements of intra-abdominal pressure were used to assess the clinical severity of strangulated hernias. Intra-abdominal pressure measurement may be used as a predictor of intestinal strangulation for patients presenting with acute abdominal compartment syndrome secondary to complicated herniation [94].

Following stabilization of the patient, the primary objective is early and definitive closure of the abdomen to minimize complications. For many patients, primary fascial closure may be possible within a few days of the initial operation. In other patients, early definitive fascial closure may not be possible. In these cases, surgeons must resort to progressive closure, in which the abdomen is incrementally closed each time the patient undergoes a subsequent surgery.

Many methods of fascial closure have been described in the medical literature [95-100].

In 2012 a retrospective analysis evaluating the use of vacuum-assisted closure and mesh-mediated fascial traction (VACM) as temporary abdominal closure was published. The study compared 50 patients treated with (VACM) and 54 using non-traction techniques (control group).

VACM resulted in a higher fascial closure rate and lower planned hernia rate than methods that did not provide fascial traction [100].

Occasionally abdominal closure is only partially achieved, resulting in large, debilitating hernias of the abdominal wall that will eventually require complex surgical repair. In these cases, delayed repair or use of biological meshes may be suggested. Bridging meshes will often result in bulging or recurrences [101]. The Italian Biological Prosthesis Working Group (IBPWG) proposed a decisional algorithm in using biological meshes to restore abdominal wall defects [60].

Another option if definitive fascial closure is not possible could be skin only closure and subsequent management of the eventration with deferred abdominal closure with synthetic meshes after hospital discharge (grade 1C recommendation).

Damage control surgery has been widely used in trauma patients and its use is rapidly expanding in the setting of Acute Care Surgery. Damage control surgery can be used in patients with strangulated obstruction and peritonitis caused by bowel perforation. Skin only closure could be an alternative for patients with failure of definitive fascia closure, reducing the risk of complications of open abdomen and abdominal compartmental syndrome [102]. Patients could be deferred for definitive abdominal closure with mesh after hospital discharge.

The component separation technique may be useful for the repair of large midline abdominal wall hernias (grade 1B recommendation).

This technique for reconstructing abdominal wall defects without the use of prosthetic material was descibed in 1990, by Ramirez et al. [103].

The technique is based on enlargement of the abdominal wall surface by translation of the muscular layers without severing the innervation and blood supply of the muscles [104].

Reherniation rates in the literature vary between 0% and 8.6%. In these series, several modifications are used, including application of prosthetic material [105-109].

In a prospective randomized trial comparing CST with bridging the defect with prosthetic material, CST was found to be superior to the insertion of prosthetic material, although a similar reherniation rate was found after a follow-up of 24 months [110].

When other means of reconstruction have already been used or are insufficient also a microvascular tensor fasciae latae (TFL) flap is a feasible option for reconstruction of exceptionally large abdominal wall defects. It can also be combined with other methods of reconstruction.

Vascularized flaps provide healthy autologous tissue coverage without implantation of foreign material at the closure site. A close collaboration between plastic and abdominal surgeons is important for this reconstruction [111].

### Antimicrobial prophylaxis

For patients with intestinal incarceration with no evidence of ischaemia and no bowel resection, short term prophylaxis is recommended.

For patients with intestinal strangulation and/or concurrent bowel resection, 48-hour antimicrobial prophylaxis is recommended. Antimicrobial therapy is recommended for patients with peritonitis (grade 2C recommendation).

In aseptic hernia repair, *Staphylococcus aureus* from the exogenous environment or the patient’s skin flora is typically the source of infection. In patients with intestinal strangulation, the surgical field may be contaminated by bacterial translocation [7,8] from intestinal villi of incarcerated ischemic bowel loops as well as by concomitant bowel resections. In patients with peritonitis both antimicrobial therapy and surgery is always recommended.

## Competing interests

The authors declare that they have no competing interests.

## Authors’ contributions

MS wrote the manuscript. All authors reviewed and approved the final manuscript.
